# Can Climatic Susceptibility Be Cured?

**Published:** 1898-01

**Authors:** E. Carleton


### "CAN CLIMATIC SUSCEPTIBILITY BE CURED?"

E. Carleton, M. D.

"The question of the evening, 'Can Climatic Susceptibility be Cured?' is closely allied to the subject of our last meeting, 'Can Susceptibility to Disease be Cured?' Hahnemann's *Organon* throws some light upon it. In the fourth American edition, Section 244, page 197, we find the following:

"'The endemic intermittent fevers of marshy districts, and countries subject to inundations are a source of much embarrassment to physicians of the prevailing school of medicine. A healthy man may, however, accustom himself in his youth to the influence of a country that is covered with morasses, and live there in perfect health, provided he confines himself to a regular mode of life, and is not assailed by want, fatigue, or destructive passions. The endemic intermittent fevers will, at farthest, attack him on his first arrival in the country; but one or two of the smallest doses of the solution of Cinchona, attenuated in a very high degree, suffice to deliver him from it promptly, if in other respects he does not depart from a strict regimen. But when a man, who takes sufficient bodily exercise, and who pursues a course every way suited to his mind and body, cannot be cured of a marsh intermittent fever by the influence of a single remedy, we may be certain that there exists within his body a psoric affection which is on the eve of developing itself, and that the intermittent fever will not yield to any other than anti-psoric treatment.'*

"'It sometimes happens that if this man quit the marshy country without delay to go and reside in another that is dry and mountainous, his health is apparently restored, and the fever leaves him, if it has not taken too deep a root—that is to say, the psora passes again to a latent state, because it had not yet reached its final degree of development; but he is not cured, nor can he enjoy perfect health, until he has made use of anti-psoric treatment.'

"This testimony in the affirmative is not to be confounded with Section 238, which should be read in connection with the three preceding sections. There Hahnemann deals with 'intermittent fevers that manifest themselves sporadically or epidemically (not the endemics of marshy districts).'

"With a larger list of important remedies to choose from than Hahnemann had (Apium-virus, Aranea-diadema, Baptisia-tinct., Eupatorium-perfoliatum, Gelsemium-sempervirens, Podophyllum-peltatum, for instance), and a wider range of potencies at command, his modern disciples accomplish more than was possible for him in this direction, and the truth of his observation is established by their experience.

"A gentleman and his wife, living not so very far from New York city, left their luxurious home and spent many months in travel, especially upon the ocean, hoping thereby to be rid of the intermittent fever, oft recurring in spite of all the arts of Old Physic. They seemed to recover health, and returned full of hope. But they had not been ashore many weeks before the

---

* Large doses of Cinchona or Sulphate of Quinine may certainly free the patient from the typical attacks of marsh intermittent fevers; but he is still unheathy in another way, and antipsorics only will effect a perfect cure.

old malady reappeared. Homœopathy then cured permanently, while their residence was unchanged from the same malarious district.

" Enlarging our field of vision, we see many people who enjoy good health in this city, but are afflicted with sore throats while sojourning in Brooklyn. They at length resort to Homœopathy, and are troubled no more. Mrs. G., wife of a lawyer, after residing for a number of years in this city in the enjoyment of health, moved to Brooklyn. She began to be subject to sore throat. The medicine of the majority was tried and found wanting. The family returned to New York, and the difficulty ceased. After a good while they ventured to move to Brooklyn again. Soon the former trouble reappeared, and was unsuccessfully combated. A friend recommended Homœopathy. These were the symptoms: Great thirst for cold water; much saliva; uvula swollen and elongated; dry, raw, rough, hot, red throat; salivary glands swollen; first appearance on right side; worse in cold air and after having been in bed a short time. A single dose of Mercury, high, made a permanent cure.

" Without doubt all agree that the cure of hay-fever is attended with difficulty. Most cases are relieved for the time being by the similar remedy, but recur the next year unless they go to Bethlehem, N. H., or some other place that gives them immunity. A portion of these same cases are cured while remaining at home. Bear in mind that some people never discover a charmed spot, as you know. It will be remembered also that many cases require antipsoric treatment, to follow that given during the acute stage. The fact is established, however, that some cases acquire, by means of Homœopathy, climatic insusceptibility to hay-fever.

" Mr. A. had for years celebrated every returning 21st day of August by starting in on a three or four weeks' tussle with hay-fever. After numerous experiments he succeeded in avoiding the attacks by going to the Adirondacks a few days ahead of time. Later on he was obliged to stay in the city all summer. The hay-fever was on hand promptly, with red, itching, smarting, sensitive, watery eyes; sneezing; profuse, acrid coryza; tingling in right nostril; occasional laryngeal cough; hoarseness; thirst; want of appetite; feverish; worse evenings and in-doors; better outdoors. Allium-cepa, 200th, in water, ended the attack in a few days, but did not prevent the same symptoms from reappearing on the next anniversary. It again relieved. Then, by searching, were found these symptoms: Hot vertex; feet hot at night, causing the patient to put them out of bed to cool off; cramps in calves when stretching. A single dose of Sulphur, millionth [B. & T.], put an end to the case.

"Change of climate brings relief to many an asthmatic individual. Numerous members of this class of sufferers have been cured by their homœopathic physicians, thus enabling them to live in the places they formerly could not endure. It is the newcomer who yields most readily to yellow fever. Presently he becomes 'acclimated,' as it is termed. That is to say, his system has in a measure become hardened and protected against the disease. And this without taking any medicine. With Homœopathy added, he becomes more strongly insured.

"Masses of evidence could be produced to sustain an affirmative answer to the question submitted, but it is to be hoped that the foregoing will be a nucleus for discussion."

DISCUSSION.

Dr. Finch—Is intermittent fever due to climate? Have we reached the cause of intermittent? New York was formerly much more troubled with it than now, except temporarily while our streets are being all torn up. Better plumbing, better drainage have brought about this change. The climate has not changed, but we have better sanitation. Hay-fever is a better instance of disease attributable to locality, but both are amenable to homœopathic treatment.

Dr. Rushmore—I am satisfied that Homœopathy enables sufferers from malaria to remain at home and become fortified against climatic influences.

Dr. Leverson said he believed that intermittent fever is due to a poison in the soil, and that infrequency of the disease is due to better sanitation; that, however, it may occur under the best sanitary conditions, because susceptibility depends upon two factors; first, intensity of the poison; secondly, individual idiosyncrasy. As an instance of the first, the west coast of Africa is deeply affected with fever, and it is hard to remove susceptibility to it. Homœopathy will alleviate considerably, but is somewhat influenced by these factors. On the other hand, a healthy, strong individual, adopting a proper hygiene, is not subject to diseases anywhere unless the poisonous conditions are very concentrated.

Dr. Finch—What constitutes climate, anything more than heat and cold, wet and dry? Reverting to the subject of hay-fever, we find that disease to be occasioned by flowering plants of various regions, and that relief is afforded by a change of abode—notably a change to mountain or sea air. Cities, *per contra*, give numerous instances of persons regularly affected with hay-fever during the warmer months. In illustration I may cite:

(1) Years ago in many parts of this city there were no sewers. Over one such undrained spot stood a frame house, in the cellar of which two or three feet of stagnant water constantly remained. The boy of the family was seized with intermittent fever. He was treated with a carefully selected remedy and removed for a time from the noxious influences to another part of the city. In a short time he was cured, really and permanently, for he was enabled to return to his home and live in perfect health, exposed to the same conditions as previously.

People are known who, out-of-doors, can stand almost any degree of cold, but in-doors are very susceptible to slight draughts or reductions of temperature. Such persons can be cured with appropriate remedies—*e. g.*, Silicea, Nux-vom., Rhus-tox., etc.

(2) The case of a gentleman residing in this city, who was annually, with clock-like regularity, seized by an attack of hay-fever. Changing his abode to an island near New York, but ten miles at sea, afforded him complete relief. The immunity lasted only so long as he remained there. Immediately upon his return to the city the disease recurred. He had not the benefit of homœopathic treatment. This simply goes to show that a change of climate alone cannot afford permanent relief to climatic susceptibility as to malaria. According to Hahnemann there is, underlying intermittent fevers, a miasm which must be removed by an antipsoric remedy before susceptibility can be cured.*

Dr. Burnet agrees in thinking that susceptibility is due to some psoric taint. When this is removed the patient is not subject to any poison, no matter how concentrated.

Dr. Clock cited three cases of persons regularly afflicted with hay-fever, who had previously been obliged to go to the seashore or mountains in order to relieve each attack; but after appropriate homœopathic treatment such excursions were unnecessary. In one case the miasm was indicated by an eruption, which appeared upon the face and which was temporarily suppressed by the use of Cuticura after shaving. Sulphur promptly and permanently relieved *all* the symptoms.

Dr. Franklin—We have to deal with the conditions of the blood and with psora. Man in his normal state is insusceptible to all diseases, no matter how malignant. Locality has no effect if a proper mode of living is maintained. Therefore we must look after the constitution and deeper conditions of the patient—*i. e.*, his state previous to exposure to the noxious influences. We have an example of this in a case of aggravated remittent fever with tendencies toward typhoid. All the family had been affected, two fatally, when the third was placed in my charge. When attempting to combat the fever alone, I met with little success; but when remedies such as Lachesis, Hepar, and particularly

---

* [NOTE by the Secretary.—"Climate, *clima*, *inclinatio coeli*, *climat*, (*κλιμα*, 'a region'). In geography the word *climate* is applied to a space on the terrestrial globe, comprised between two circles parallel to the equator, and arbitrarily measured according to the length of the days. In a hygienic point of view it means, since Hippocrates, a country or region, which may differ from another in respect to season, qualities of the soil, heat of the atmosphere, etc. Climate, indeed, embraces, in a general manner, all the physical circumstances belonging to each region—circumstances which exert considerable influence on living beings. The dark complexion of the inhabitants of the torrid zone is easily distinguishable from the paleness of those of the frigid zone—so are the diseases. They are all modified, more or less, by climate or locality. Hot climates predispose to abdominal complications in febrile affections; cold climates to thoracic, etc.—DUNGLISON."]

Silicea, were given for the general constitutional condition, there was a decided abatement of the fever (acute condition), besides great general improvement. Hence we may conclude that only general, systemic conditions render acute diseases possible.

Dr. Pease agrees with Dr. Finch that change of abode can only alleviate or temporarily remove susceptibility to local climatic conditions; and, in support of his view, cities the case of a lady who was always subject to aphonia when in New York. This would disappear when she left the city and vicinity, only to recur promptly upon her return.

Dr. Talcott—Statistics show that the Hebrew race is the only one which can and does live in all climates. This is perhaps because the Hebrews adapt themselves to their surroundings. Other races which migrate do not thrive—*e. g.*, the English in India. Proper and wise medication would, however, enable almost any one to live in a foreign climate, but in lack of that few of us have sufficient constitution to abide the change. After several generations, by a kind of evolutionary process, we can resist any climatic condition, but many fall by the wayside.

Having tried it upon my own person, I have reached the conclusion that climate alone can cure no one. If a person would be cured, it is by some appropriate medicine. Many chronic cases try a change of climate, and are for a time improved, but upon return to the old surroundings they promptly relapse into their former condition. Several cases were mentioned in illustration.

Dr. Carleton—In the absence of Dr. Fincke it devolves upon me to close the discussion. Both papers that have been read favor the affirmative side of the question, and both cite Section 244 of Hahnemann's *Organon* in their support. In that Section Hahnemann specifies "the endemic intermittent fevers of marshy districts." In the nine Sections just preceding, he is careful to say "Intermittent fevers that manifest themselves sporadically or epidemically (not the endemics of marshy districts)." It is necessary to bear this in mind, to avoid a seeming contradiction. The first part of Section 241 declares that "epidemical intermittent fevers, in places where none are endemic, have the nature of chronic diseases, composed of individual, acute paroxysms."

Clearly, the endemic marsh intermittent mentioned by Hahnemann is a product of climate, as that word is commonly used by physicians. He says that susceptibility to it can be cured; and that has been the experience and observation of his followers ever since.

African coast fevers are serious. Homœopathy has never had a fair chance at them. In the opinion of the speaker, they are amenable to the similar remedy. The question of the Briton in India is an interesting one. He tries to take his British habits with him; he will not bend to the local customs; will wear heavy clothing, drink beer, and live with the habits of a Northern climate. In consequence, his liver becomes enlarged, his system clogged, and he has to return to his native land. If he marries and raises a family, the latter are soon forced to seek a more suitable climate. Only those who, like Livingstone in Africa, adapt themselves to the local conditions and customs, can live and procreate.

### CLINICAL CASES.

Dr. Leverson presented a very full account of a case of suppressed gonorrhœa with supposed sycosis and iodism, over which he solicited discussion. Thuja had occurred to him as an appropriate remedy to be given after the effects of the iodide had somewhat worn away

Dr. Vondergoltz said that in his experience it had been better not to wait, but to give the selected remedy immediately and in high potency.

Dr. Gillingham thought that the symptoms indicated that Pulsatilla might be needed before the Thuja.

Dr. Krause thought that there was little hope of bringing out, under the influence of Thuja, the suppressed discharge, and that it would be more advisable to study the symptoms as they exist at present, and to prescribe in accordance.

Dr. Carleton, quoting a similar condition from Lippe's *Materia Medica*, showed that Hepar-sulph. followed by Nitric-acid, and these possibly in turn by Pulsatilla and finally Thuja, seemed to be indicated.

Dr. Rushmore—Hahnemann, during the early years of his practice, tried, when he found a psoric condition, to bring back the eruption. Later in life he states that it is not necessary nor always possible.

Dr. Franklin—Case I. Well-marked phthisis in a patient of forty. All the usual signs, including those of cavities, were present. Physicians of the old school had pronounced the case hopeless under any treatment. Nevertheless, a single dose of Sulphur 200 was given. After a short time, an eruption came on. It began with isolated yellowish crusts and underlying ulcers. These became confluent until nearly the whole body surface was covered. Then the cough, dyspnœa, and chest symptoms subsided. The eruption soon followed, leaving the patient perfectly well. So he continued for years, and finally died from Morphine poisoning.

Case II—Otorrhœa, resulting from the allopathic treatment of eczema of the scalp. Under the properly-selected remedy, it ran a retrograde course with final cure.

Owing to the late hour and the large amount of clinical matter remaining, upon the motion of Dr. Finch, that the latter be carried over to the next meeting, the meeting was adjourned.

WALTER S. GOULD,
*Secretary pro tem.*

